# COVID-19 Classification from Chest X-Ray Images: A Framework of Deep Explainable Artificial Intelligence

**DOI:** 10.1155/2022/4254631

**Published:** 2022-07-14

**Authors:** Muhammad Attique Khan, Marium Azhar, Kainat Ibrar, Abdullah Alqahtani, Shtwai Alsubai, Adel Binbusayyis, Ye Jin Kim, Byoungchol Chang

**Affiliations:** ^1^Department of Computer Science, HITEC University, Taxila, Pakistan; ^2^Department of Computer Science, University of Wah, Wah Cantt, Pakistan; ^3^College of Computer Engineering and Sciences, Prince Sattam Bin Abdulaziz University, Al-Kharj, Saudi Arabia; ^4^Department of Computer Science, Hanyang University, Seoul 04763, Republic of Korea; ^5^Center for Computational Social Science, Hanyang University, Seoul 04763, Republic of Korea

## Abstract

COVID-19 detection and classification using chest X-ray images is a current hot research topic based on the important application known as medical image analysis. To halt the spread of COVID-19, it is critical to identify the infection as soon as possible. Due to time constraints and the expertise of radiologists, manually diagnosing this infection from chest X-ray images is a difficult and time-consuming process. Artificial intelligence techniques have had a significant impact on medical image analysis and have also introduced several techniques for COVID-19 diagnosis. Deep learning and explainable AI have shown significant popularity among AL techniques for COVID-19 detection and classification. In this work, we propose a deep learning and explainable AI technique for the diagnosis and classification of COVID-19 using chest X-ray images. Initially, a hybrid contrast enhancement technique is proposed and applied to the original images that are later utilized for the training of two modified deep learning models. The deep transfer learning concept is selected for the training of pretrained modified models that are later employed for feature extraction. Features of both deep models are fused using improved canonical correlation analysis that is further optimized using a hybrid algorithm named Whale-Elephant Herding. Through this algorithm, the best features are selected and classified using an extreme learning machine (ELM). Moreover, the modified deep models are utilized for Grad-CAM visualization. The experimental process was conducted on three publicly available datasets and achieved accuracies of 99.1, 98.2, and 96.7%, respectively. Moreover, the ablation study was performed and showed that the proposed accuracy is better than the other methods.

## 1. Introduction

Due to the emergent spread of coronavirus infection, the COVID-19 pandemic has become a worldwide challenge since December 2019 [1]. It was recognized as a “Public Health Emergency of International Concern (PHEIC)” by the “World Health Organization (WHO)” that had a potential impact on billions of lives. This virus potentially originated in Wuhan, which is the capital city of Central China, and appeared in the form of severe atypical pneumonia affecting the lower respiratory tract that often leads to the death of a person [2]. Up till now, the total number of worldwide active cases reported till September 2021 on a daily basis is 353,936, the death toll till September on a daily basis is 5784, and an estimated amount of active cases and the death rate from the beginning of pandemic till September 2021 is 229,354,842 and 4,706,699, respectively, till 19 September 2021 [3, 4]. With this virus, 221 countries and their zones have been affected since 2019, continuing to spread globally across the world, including a wide range of countries in which the top five countries with the highest reported coronavirus cases are the USA, India, Brazil, UK, and Russia. The USA lies above the mentioned top five countries with the highest number of active cases, that is, 402,908,749, since its beginning.

Coronavirus infection is thought to be highly contagious. To combat the spread of infection, it should be detected early, and patients should be quarantined [5]. The gold standard for detecting coronavirus infection is reverse transcription polymerase chain reaction (RT-PCR) [6, 7]. RT-PCR comprises detecting viral RNA from a nasopharyngeal swab. The major limitation of the RT-PCR test is that it has less sensitivity, due to which it is not efficient to detect the rate of positive cases rapidly. This test requires more time to get the results; moreover, the availability of material required is limited in the health sector [6]. To overcome this limitation, biomedical imaging-based methods such as chest X-ray images, radio images, or computed tomography (CT) scanners could be used for rapid screening [8, 9]. Detection of coronavirus infection at the initial stage through biomedical imaging can avoid the spread of this contagious disease [10]. Automated COVID-19 classification techniques have been preferred over manual techniques, which usually are followed by a preprocessing, segmentation, selection, and classification phase. As a consequence, there is a need to introduce an artificial intelligence- (AI-) based decision support system. This system segments the infection along with detection at the lung level through images. Deep learning plays a vital role in the classification of COVID-19 [11]. Biomedical imaging-based CNN architecture turned out to be a reliable technique [12] for image segmentation as well as image classification [13, 14]. Apart from prior stated models, InceptionResNetV2, ResNet50, DenseNet20, VGG19, MobileNetV2, NasNetMobile, and ResNet15V2 have been widely used in medical imaging [15, 16].

**Figure 1:**
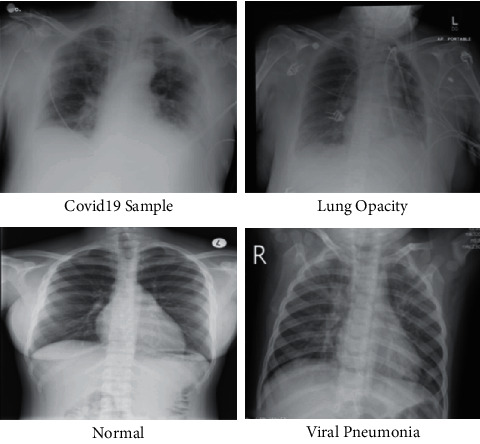
Sample image of COVID-19 Radiography dataset.

Artificial intelligence has become an emergent field by using its various techniques [17], such as deep neural networks, to solve a variety of problems, which are image classification, object detection, drug interaction, medical imaging [18, 19], and speech recognition [20–22]. More precisely, convolutional neural networks (CNN) showed promising results in the field of image processing [23]. Enormous research studies presented the robustness of these techniques for image segmentation [24]. Explainable artificial intelligence (XAI) is basically the integration of multiple AI models into an ergonomic GUI to assist radiologists in decision-making in order to improve understanding of COVID-19 [25]. The recent studies faced several challenges for the accurate classification of COVID-19 with normal chest X-ray images. The first challenge is multiclass classification such as COVID-19, viral pneumonia, lung opacity, and normal images. Visually, these images are shown in Figure 1. From this figure, it is observed that the similarity among each image is very high, and it is a chance of misleading the correct classification accuracy. The second challenge is redundant and irrelevant feature extraction that not only degrades the classification accuracy but also increases the computational time. In this work, we proposed a new framework based on deep learning and explainable AI for COVID-19 classification. Our major contributions are listed as follows:

- We proposed a hybrid contrast enhancement technique based on the fusion of Weiner filtering, global information, and box filtering.
- Two deep learning pretrained models are modified and trained through transfer learning on an enhanced dataset. Later, Grad-CAM-based visualization is performed, and at the same time, features are extracted from the global average pooling layer.
- An improved version of the discriminant canonical correlation analysis-based method is implemented for feature fusion.
- A hybrid feature optimization algorithm is proposed named Whale-Elephant Herding for the best features selection. The final features are classified using an extreme learning machine (ELM).

The rest of the paper is organized in the following order: Section 2 describes the related work in which recent techniques are discussed. The proposed methodology is presented in Section 3. Results of the proposed methodology are discussed in Section 4. Finally, Section 5 concludes the paper.

## 2. Related Work

The outbreak of coronavirus all over the world was inciting pain and caused an alarming situation everywhere in a short time. For early detection, various traditional methods are used, such as reverse transcription polymerase chain reaction (RT-PCR), CT scan, and X-ray imaging. But the detection of coronavirus using these traditional methods is time taking and not so accurate. Recently, various machine learning, artificial intelligence, and deep learning-based methods have been presented for early detection of this disease to overcome the rapidly increasing death rate. Wu et al. [26] introduced a joint classification and segmentation technique for real-time and explainable COVID-19 detection using chest CT on the COVID-CS dataset. Reis and Oliveira-Esquerre [27] presented an explainable AI-based method for COVID-19 detection using blood cell count. Five AI-based algorithms were used for evaluation, and a Bayesian optimization was applied for hyperparameters tuning. Karim et al. [28] presented an automated method for COVID-19 detection in CXR images based on an explainable DNN method. In this method, class distinguishing regions are highlighted by gradient-guided class activation maps and by using layer-wise relevance propagation. Further human-illustratable description for prediction is used. In [29, 30], an automated method for the detection of coronavirus in chest CT images using DCNN models was introduced. A transfer learning technique with custom-sized input is used in the models for best accuracy results. For better illustration, visualization methods are used for the visual explanation of predicted models. Soares et al. [31] presented an explainable-by-design method for COVID-19 classification using a CT scan. In this approach, integrated segmentation with SLIC superpixel is used for better performance and illustration. Shi et al. [32] presented an explainable attention transfer classification method using a knowledge distillation network for COVID-19 detection. A teacher network is used to extract global features and uses the deformable attention model for clear depiction. Then, image fusion is applied to combine extracted features from global and irregular-shaped scratch regions. Ahsan et al. [33] presented an automated model for COVID-19 detection using six deep CNN models and using different CT scans and chest X-ray images for identification. The modified MobileNetV2 gives promising results as compared to other models. Singh et al. [34] suggested a deep learning-based method for COVID-19 detection. In this approach, image enhancement, image segmentation, and modified stack ensemble model with Naïve Bayes as metalearner for classification are used. Moreover, explainability is applied using Grand-Cam visualization, and various existing GAN architectures are used to create realistic copied samples to cope with limited sample numbers. Mahmoudi et al. [35] applied explainable DL models for the classification and segmentation of COVID-19 images by using different X-ray and CT scan images. Lo and Yin [36] introduced an interaction-based CNN model for COVID-19 detection using chest X-ray images.

Moreover, Pennisi et al. [25] adopted the AI-based technique for COVID-19 classification along with the lungs' lesion category. They first presented a model to identify the lung parenchyma and lobes and then combined two networks, including segmentation and classification, for COVID-19 classification and lesion categorization. Their dataset included 166 individuals' CT scans, 72 of them were COVID positive, and 35 were interstitial but COVID negative. Similarly, Palatnik et al. [37] identified the issue of usage of false or doubtful artifacts in dataset images by classifiers; they suggested a new AI-based technique for COVID-19 classification on the COVID-CT dataset [38]. They combined multiple AI algorithms, including Grad-CAM, lime, RISE, square grid, and direct gradient approaches, to compare their results and evaluated the exposed biases for the studied classifier. Researchers in [39] highlighted the issue of robustness in the AI systems due to undesired learned shortcuts. Their scheme provided significant proof that explainable AI should be seen as a prerequisite to deploying machine learning-based healthcare models. Another study made by [40] presented promising quantification and qualitative visualizations by proposing an XAI-equipped classifier for COVID-19 detection. They tested their strategic module on a privately collected dataset from the local hospitals, then ensured its efficiency by training the publicly available CC-CCII dataset with 2,034 CT volumes, and achieved favorable results. The previous research concentrated on traditional machine learning and deep learning techniques for COVID-19 classification. They improved their accuracies on some datasets, but they ran into problems with low contrast images, redundant feature extraction, and selecting a relevant classifier. The preprocessing step for improved contrast of input images was not considered in the preceding studies. They also skipped the step of boosting the best features. We concentrated on contrast stretching, feature optimization, and explainable AI in this work to achieve better results on selected datasets.

## 3. Materials and Methods

**Figure 2:**
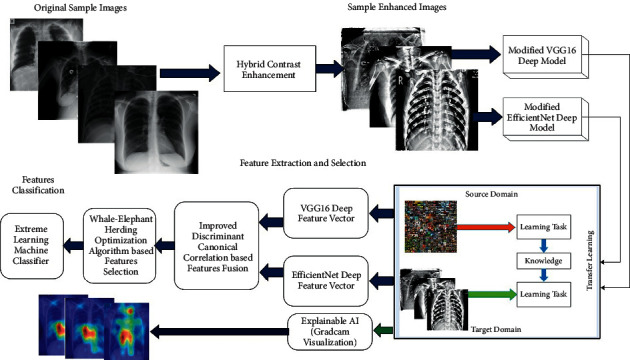
Main architecture of proposed framework for classification of COVID-19 infected Chest X-ray images.

The proposed deep learning and explainable AI-based framework for COVID-19 classification is presented in this section.  Figure 2 illustrates the overall architecture of the proposed methodology for COVID-19 classification. Initially, a hybrid contrast enhancement technique is proposed and applied to the original images that are later utilized for the training of two modified deep learning models. The deep transfer learning concept is used for the training of pretrained modified models that are later employed for feature extraction. Features of both deep models are fused using improved canonical correlation analysis that is further optimized using a hybrid algorithm named Whale-Elephant Herding. Through this algorithm, the best features are selected and classified using an extreme learning machine (ELM). The description of each substep is given as follows.

### 3.1. Hybrid Contrast Stretching

The purpose of the contrast stretching technique is to improve the visibility of an image in terms of pixel refinement. In this work, we proposed a hybrid contrast stretching technique based on the fusion of three filtering methods such as Weiner filter, global information improvement, and box filtering. The purpose of these methods is to get a clearer image for better feature extraction in the next step. Consider that we have a database denoted by Δ having *n* numbers of images, *f*_*i*_(*u*, *v*) represents the image in the database, and $\tilde{f_{i}}\left(u,v\right)$ denotes the final resultant image, respectively. The Weiner filter is initially applied to each image. This filtering technique is ideal in terms of the mean square method as it helps to minimalize mean square error in inverse filtering and noise smoothing process. After taking the input image, blurring operation and deconvolution are performed by using a low pass filter and inverse filtering, respectively, which finally results in the removal of noise using compression operation.

(1) $$\begin{array}{c} H=\frac{1}{16}\left[\begin{array}{ccc} 1 & 1 & 1\\ 1 & 1 & 1\\ 1 & 1 & 1\\ 1 & 1 & 1 \end{array}\right],\\ J\left(a,b\right)=\frac{H\left(a,b\right)f_{x}\left(a,b\right)}{{\left|H\left(a,b\right)\right|}^{2}f_{x}\left(a,b\right)+f_{y}\left(a,b\right)}, \end{array}$$

where *H*(*a*, *b*) represents the Fourier transform of the point spread function, *f*_*x*_(*a*, *b*) represents the power spectrum of the pixel process obtained by taking the Fourier transform of the pixels autocorrelation, and *f*_*y*_(*a*, *b*) denotes the power spectrum of the noise process obtained by taking the Fourier transform of the noise autocorrelation. The global information of resultant *J*(*a*, *b*) is increased by employing the following equation:

(2) $$\begin{array}{c} g\left(u,v\right)=J\left(a,b\right)-\left(J\left(a,b\right){}^{\circ}b\right), \end{array}$$

where *g*(*u*, *v*) denotes the global pixels enhanced image, *b* is an increasing variable, and value is computed using

(3) $$\begin{array}{c} b=\text{MAX}\left(J\left(a,b\right)\right). \end{array}$$

**Figure 3:**
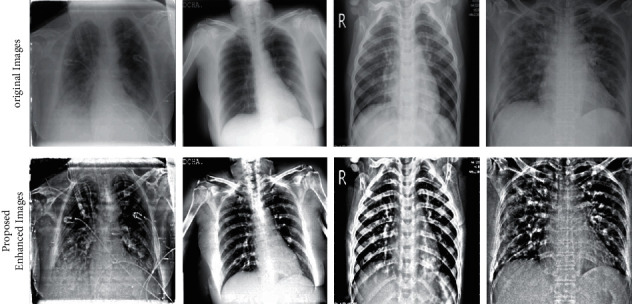
Sample images of the proposed contrast enhancement technique.

After that, a box filter is applied to the resultant image *g*(*u*, *v*). In box filtering, the average of neighboring pixels is used to filter noise from images. Mathematically, it is formulated as follows:

(4) $$\begin{array}{c} B_{x,y}=\frac{\sum_{i=x-J}^{x+J}\sum_{j=y-K}^{Y+K}{\tilde{g}}_{ij}}{\left(2J+1\right)\left(2K+1\right)}, \end{array}$$

where *g*_*ij*_ denotes the 8–16-bit sample of *g*(*u*, *v*) image and $\tilde{g}$ is the box filter that provides sample *B*_*x*,*y*_ of *g*(*u*, *v*) image. The box constant *H*_*jk*_^*JK*^ within (2*J*+1)(2*K*+1) is given as follows:

(5) $$\begin{array}{c} H_{jk}^{JK}=\frac{1}{\left(2J+1\right)\left(2K+1\right)}, \end{array}$$

where *B*_*x*,*y*_ is the final enhanced image, and visually, it is illustrated in Figure 3. These resultant images are further utilized for the training of selected deep learning models.

### 3.2. Deep Learning Models

#### 3.2.1. EfficientNet

EfficientNet is a convolutional network (ConvNet) scaling technique that utilizes the simple yet potent compound scaling method. EfficientNet enables us to scale up a baseline ConvNet to any desired level of computational resources while sustaining the original model efficiency [47, 48]. It is based on the key principle of uniformly scaling the network depth, width, and resolution with a set of constant scaling factors. To understand this, let us assume that we want to increase the resource constraint of our network with a factor of 2^*x*^. To achieve this increase, we require to scale our network depth by *μ*^*x*^, width by *ω*^*x*^, and resolution by *σ*^*x*^, where *μ*, *ω*, and *σ* are constant scaling factors determined by searching the original model with a small grid. It uses a compound coefficient *ε* to uniformly scale the network resources in all three dimensions characterized by these formulations as depth: D =  *μ*^*ε*^, width: *W* =  *ω*^*ε*^, and resolution: *R* =  *σ*^*ε*^. EfficientNet consists of a series of efficient family models called EfficientNet-B0 to EfficientNet-B7, and each contains a different set of parameters and FLOPS ranging from 5.3 M to 66M and 0.39 B to 37B, respectively. The accuracy and effectiveness of EfficientNet are better than all existing CNN models such as AlexNet, ImageNet, GoogleNet, and MobileNetV2 [41].

#### 3.2.2. VGG16

VGG16 deep model was originally trained on the ImageNet dataset and gave an excellent performance for both large and smaller datasets. This network includes 16 convolutional layers and has a small receptive field of 3 × 3. It contains 5 max-pooling layers of size 2 × 2 and three fully connected layers and one Softmax classifier. The activation layer named ReLU is applied to all hidden layers.

#### 3.2.3. Modification

**Figure 4:**
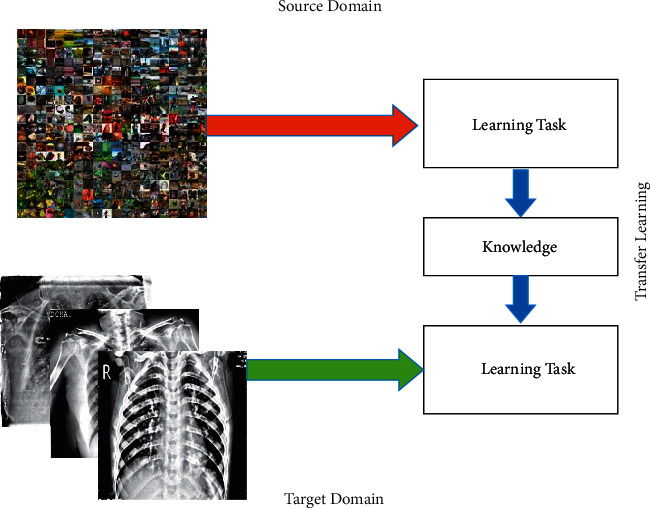
Deep transfer learning-based model training.

In this work, we modified both deep learning models in terms of layer removal and the addition of new layers. In the EfficientNet deep model, the last FC, classification, and Softmax layer are removed, and three new layers, New-FC, New-Classification, and New-Softmax, are added. After the addition of new layers, hyperparameters are initialized: learning rate is 0.002, minibatch size is 32, momentum is 0.7, dropout factor is 0.5, and training function is Adam. After that, train this modified model using a transfer learning concept and obtain a new model, whereas the freezing layers considered only 20% (starting at 20%). The process of transfer learning is illustrated in Figure 4. The modified trained model was considered for EfficientNet feature extraction. Similarly, in VGG16, the last FC layer (FC8), classification layer, and Softmax layer were removed, and three new layers, New-FC8, New-ClassificationOut, and New-SoftVgg, were added. Then, this modified model was trained using the transfer learning process, whereas the same hyperparameters were considered (as mentioned above) for a training purpose. In the last, a new modified VGG16 model is obtained, and deep features are extracted.

#### 3.2.4. Features Extraction

Features are extracted from the modified trained EfficientNet and VGG16 deep models. For modified EfficientNet, activation is applied to the global average pooling layer, and features of dimensional *N* × 1280are extracted. Similarly, features of VGG16 deep models are extracted from FC layer 7, and a feature vector of dimension *N* × 4096 is obtained. The extracted feature vectors are fused in one matrix for better information and improved accuracy.

### 3.3. Improved DCCA-Based Features Fusion

Features are fused using an improved discriminant canonical correlation analysis-based technique, and a new improved feature vector of dimension *N* × 2864 is obtained. In this approach, initially, mean padding is performed, and after that, DCCA formulation is applied to get a feature vector. The major improvement in DCCA is the mean padding, and a threshold function for important feature addition in the fusion process is defined. The DCCA [34] belongs to the category of dimensionality reduction methods that work on the basis of supervised learning that examines the linear relationships among a pair of sets of random vectors. {*u*_*i*_, *v*_*i*_}_*i*=1_^*n*^*ϵR*^*p*^ × *R*^*q*^ represents *n* pairs of samples that are normalized based on mean computation, where *c* represents the class number. DCCA often encounters optimization problems that can be solved as follows:

(6) $$\begin{array}{c} \left\{\begin{array}{cc} \max & \rho \left(\alpha ,\beta \right)=\alpha^{T}\text{UAV}\beta ,\\ s.t. & \begin{array}{c} \alpha^{T}S_{uu}\alpha =1,\\ \beta^{T}S_{vv}\beta =1, \end{array} \end{array}\right. \end{array}$$

where *Z* = diag (1_*n*_1_×*n*_1__, 1_*n*_2_×*n*_2__,…, 1_*n*×*n*_*c*__) and *n*_*k*_ represents the total amount of training set of a specified class *k*, where ∑_*k*=1_^*c*^*n*_*k*_=*n*. To resolve the optimization problem, the generalized eigenvalue problem is used on the basis Lagrange multiplier theorem:

(7) $$\begin{array}{c} \left[\begin{array}{cc} 0 & \text{UAV}\\ {\left(\text{UAV}\right)}^{T} & 0 \end{array}\right]\left[\begin{array}{c} \alpha \\ \beta \end{array}\right]=\lambda \left[\begin{array}{cc} S_{uu} & 0\\ 0 & S_{vv} \end{array}\right]\left[\begin{array}{c} \alpha \\ \beta \end{array}\right], \end{array}$$

where *λ* stands for Lagrange multiplier. The resultant fused vector obtained based on equation (7) is passed to a threshold function to get the improved vector.

(8) $$\begin{array}{c} \text{Vector}=\left\{\begin{array}{c} \text{Fused}\left(\lambda \right)\text{ for feat}\geq 0.5,\\ \text{Drop,Elsewhere}. \end{array}\right. \end{array}$$

### 3.4. Whale-Elephant Herding Optimization

Features optimization is the process of obtaining the best features and passed to the machine learning techniques for better classification accuracy. Moreover, the optimization step's main aim is to reduce the computational time of the entire developed framework. In this work, we proposed a Hybrid Whale-Elephant Herding (HWEH) algorithm for best feature selection. The proposed algorithm returned a feature vector of dimension *N* × 824.

The original whale optimization algorithm is introduced by Mirjalili and Lewis [42]. The main concept was derived by observing the hunting trait of the humpback whales. Naturally, the whales create bubbles while swimming spirally in the direction of the prey. Based on its metaheuristic characteristic, WOA has two distinct stages: (i) exploitation and (ii) exploration. The simulation of the exploitation stage is carried out by surrounding the prey spirally and moving in its direction.

(9) $$\begin{array}{c} E=\left|B.\overset{⟶}{Y}*\left(i\right)-\overset{⟶}{Y}\left(t\right)\right|,\\ \overset{⟶}{Y}\left(i+1\right)=\overset{⟶}{Y}*\left(i\right)-\overset{⟶}{X}\cdot E, \end{array}$$

where *i* represents current iteration and *Y* and *Y*^*∗*^ indicate the current and best whales, that is, solution, respectively. The coefficient vectors $\overset{⟶}{X}$ and $\overset{⟶}{B}$ are defined as follows:

(10) $$\begin{array}{c} \overset{⟶}{X}=2\overset{⟶}{x}\cdot \overset{⟶}{r}-\overset{⟶}{x},\\ \overset{⟶}{B}=2\cdot \overset{⟶}{r}. \end{array}$$

The value of $\overset{⟶}{x}$ decreases from 2 to 0 in a linear manner along with iterations and $\overset{⟶}{r}$ indicates a random vector in the range of [0,1].

(11) $$\begin{array}{c} \text{x}=2\left(1-\frac{i}{I}\right). \end{array}$$

The spiral-shaped path is modeled by the spiral rule as follows:

(12) $$\begin{array}{c} \overset{⟶}{Y}\left(i+1\right)=E\prime \cdot e^{bl}\cdot \;\cos\left(2\pi l\right)+\overset{⟶}{Y}*\left(i\right),\\ E^{\prime }=\left|Β\cdot \overset{⟶}{Y}*\left(i\right)-\overset{⟶}{Y}\left(i\right)\right|, \end{array}$$

where E′ indicates the distance between the ith search agent and $\overset{⟶}{Y}*\left(i\right)$ and *b* is a content where *l* is a random number in the range of [−1,1]. The selection between the shrinking encircling and the spiral shape path is assessed by the probability of 40% instead of 50%.

(13) $$\begin{array}{c} \overset{⟶}{Y}\left(i+1\right)=\left\{\begin{array}{c} \text{Shrinking Encircling using Eq}.\left(12\right)p<0.4,\\ \text{Sprial Shaped Path using Eq}.\left(16\right)p\geq .5, \end{array}\right. \end{array}$$

where *p* is any random number in a range of [0, 1]. For the exploration phase of WOA, the positions of the current whales are modified by randomly selecting a search agent from the population in spite of modifying their positions according to the best solution to reduce the chances of the solution getting trapped in local optima. This approach is represented mathematically as follows:

(14) $$\begin{array}{c} Ε=\left|Β{\overset{⟶}{Y}}_{rand}\left(i\right)-\overset{⟶}{Y}\left(i\right)\right|,\\ \overset{⟶}{Y}\left(i+1\right)={\overset{⟶}{Y}}_{rand}\left(i\right)-\overset{⟶}{X}\cdot Ε, \end{array}$$

where ${\overset{⟶}{Y}}_{\text{rand}}$ is a randomly selected search agent and $\overset{⟶}{X}$ represents a vector that is assigned any random value from range [>−1,<1]. The output of equation (14) is considered as the input of the Elephant Herding algorithm. The Elephant Herding Optimization (EHO) is an intelligent swarm-based metaheuristic optimization algorithm that was proposed by getting moved by the herding manner of the elephants [43]. This algorithm works in three steps: (i) the flock of elephants formed of a smaller group, also known as clans, where each clan holds a definite amount of elephants; (ii) the female elephant in each clan, also called matriarch, acts as a leader who is responsible for commanding the whole clan and all other elephants move together under her governance, and (iii) in each generation, a defined number of elephants with poor performance are required to leave the clan. Mathematically, this process is defined as follows.

#### 3.4.1. Clan Updating Operator

As stated prior, each flock of elephants comprises a smaller group, also known as clans, where each clan holds a definite amount of elephants. All elephants in a clan move together under the leadership of a female elephant, also called matriarch *Y*_*kbest*,*c*_, which influences their next position formulated as follows:

(15) $$\begin{array}{c} Y_{m,c}^{a+1}=Y_{m,c}^{a}+\alpha \times \left(Y_{k\text{best},c}^{a}-Y_{m,c}^{a}\right)\times r, \end{array}$$

where *Y*_*m*,*c*_^*a*+1^ and *Y*_*m*,*c*_^*a*^ represent the newly updated position and old position of *m*th number of elephants (*m* = 1, 2, 3, 4,…, *e*) and *c*th (*c* = 1, 2, 3, 4,…, *n*) number of clans, respectively. The impact of the matriarch who is the fittest female elephant individual of the clan (Y_kbest, c_) on existing elephant *Y*_*m*,*c*_ is determined by the scaling factor *α* ∈ [0,1]; a uniform distribution has been used, which is represented by *r,* also known as a random number, *r* ∈ [0,1]. The value of the matriarch in the respective clan cannot be updated by means of an equation such as *Y*_*m*,*c*_^*a*^  =  *Y*_kbest,*c*_^*a*^. The updation of the fittest one is formulated as follows:

(16) $$\begin{array}{c} Y_{k\text{best},c}^{a+1}=\beta \times Y_{\text{center},c}^{a}, \end{array}$$

where scaling factor is represented by *β* and *β*∈ [0,1] defines the impact of *Y*_center,*c*_^*a*^ on *Y*_kbest,*c*_^*a*+1^. *Y*_center,*c*_^*a*^ represents clan center, and *Y*_*kbest*,*c*_^*a*+1^ represents clan leader's new position. Mathematically, the clan center value is computed by

(17) $$\begin{array}{c} Y_{\text{center},c}=\frac{1}{e}\times \sum_{z=1}^{e}Y_{m,c}. \end{array}$$

#### 3.4.2. Separating Operator

**Figure 5:**
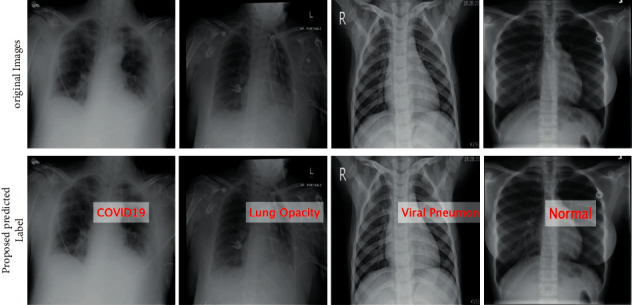
Labeled results of the proposed method.

In this operation, the replacement of the worst individuals is performed by using

(18) $$\begin{array}{c} Y_{\text{kworst},c}=Y_{\text{MIN}}\left(Y_{\text{MAX}}-Y_{\text{MIN}}+1\right)\times r, \end{array}$$

where Y_MIN_ and Y_MAX_ denote the lower bound and upper bound, respectively, of the individual position of the elephant. Y_kworst,c_ represents the worst individual elephant of the *c*th clan, which is to be swapped by the arbitrarily initialized individual, and *r* embodies uniform distribution in the range of [0, 1] where *r* ∈ [0, 1]. In Elephant Herding Optimization, the clan updating operation does not allow the clan head to explore outside the clan as their position is restructured according to the clan center. The neural network is utilized in this work for fitness function, and fitness is computed based on the mean square error rate. The best resultant vector is passed to the extreme learning machine (ELM) classifier for final results. The proposed labeled results are shown in Figure 5.

### 3.5. Grad-CAM Visualization

Gradient weighted Class Activation Mapping (Grand-CAM) [44] is a class-classification localization method that is used in with various CNN-based models without architectural changes for the generation of visual description. In CNN modeling, the convolutional features hold spatial information that is lost in a fully connected layer. So, the last convolutional layers are expected to have the best conciliation between high-level semantics and exhaustive spatial information. It uses gradient information of the last convolutional layer of the CNN model to recognize the position of each neuron for making a decision. In this work, we utilized the modified pretrained models and performed Grad-CAM visualization.

To get class-classification localization map Grad-CAM *G*_Grad−CAM_^*C*^ ∈ *N*^*m*×*n*^ of width *m* and height *n* for any class *C*, firstly, gradient of the score for class *C* is computed, and *x*^*C*^ is used before Softmax layer, according to feature maps *F*^*l*^ of a convolutional layer, that is, *∂x*^*C*^/*∂F*^*l*^. These gradients are global average-pooled to get the neuron weights *β*_*l*_^*C*^.

(19) $$\begin{array}{c} \beta_{l}^{C}=\frac{1}{Y}\sum_{a}^{n}\sum_{b}^{n}\frac{\partial x^{C}}{\partial F^{l}}. \end{array}$$

These weights represented by *β*_*l*_^*C*^ signify a partial linearization of downstream deep network from *F* and get the significance of feature map *l* for a target class *C*. Generally, there is no need for *x*^*C*^ for class score but can be any differentiable activation. Grad-CAM heatmap is a weighted combination of advancing activation features patterns, which is followed by using ReLU to compute

(20) $$\begin{array}{c} G_{\text{Grad}-\text{CAM}}^{C}=\text{ReLu}\left(\sum_{l}^{n}\beta_{l}^{C}F^{l}\right). \end{array}$$

**Figure 6:**
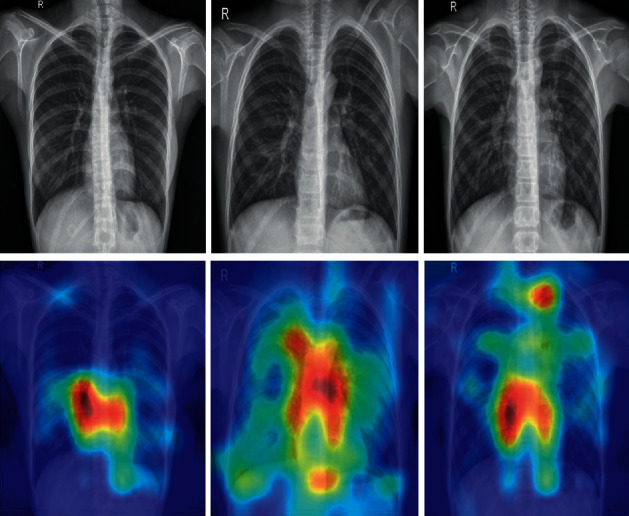
Grad-CAM-based visualization.

This obtained outcomes in an abrasive heatmap, which is normalized for visualization [46]. Visually, it is shown in Figure 6.

## 4. Results and Analysis

The experimental process of the proposed framework is presented in this section. The proposed framework is evaluated on three publicly Kaggle COVID datasets such as COVID-19 Radiography dataset (https://www.kaggle.com/tawsifurrahman/covid19-radiography-database), Covid-GAN and Covid-Net mini Chest X-ray dataset (https://www.kaggle.com/yash612/covidnet-mini-and-gan-enerated-chest-xray), and Chest X-ray dataset (https://www.kaggle.com/jtiptj/chest-xray-pneumoniacovid19tuberculosis), respectively. COVID-19 Radiography database consists of positive COVID-19 chest X-ray images along with viral pneumonia, lung opacity, and normal classes. This dataset contains 3616 images of COVID-19, 6012 lung opacity images, 10192 normal images, and 1345 viral pneumonia images. Covid-GAN and Covid-Net mini Chest X-ray dataset consists of 461 COVID-19 images, 1575 normal images, and 4481 pneumonia images. Chest X-ray dataset consists of four types of categories such as 566 COVID-19 images, 1575 normal images, 4265 pneumonia images, and 491 tuberculosis images, respectively. The summary of each dataset is given in Table 1. Each dataset is divided into a ratio of 50 : 50 for training data and testing data. The 10-fold cross-validation is used for each set. Several classifiers are selected for the classification process to compare the results with the main classifier such as ELM. The performance of each classifier is a measure based on the average accuracy and computational time. The entire framework is simulated on MATLAB2021b using Personal Workstation with 16 GB RAM and 8 GB graphics card.

### 4.1. Detailed Results

**Figure 7:**
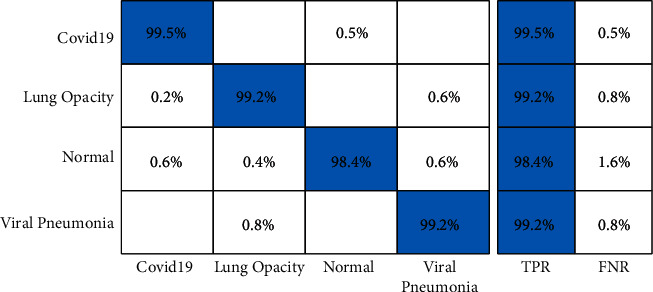
Confusion matrix of ELM classifier on COVID-19 Radiography database using the proposed framework.

The proposed framework is tested in several middle steps such as (i) EfficientNet deep features extraction (EffNet); (ii) VGG19 deep features (VGG16); (iii) selected EfficientNet deep features (SL EffNet); (iv) selected VGG19 deep features (SL VGG16); and (v) fusion of selected features using DCCA-based approach (Proposed).  Table 2 presents the classification results of the COVID-19 Radiography database on all middle steps. ELM classifier gives better results than Softmax, Naïve Bayes, and MCSVM. For ELM classifier, achieved accuracy on EffNet is 92.8%, VGG16 is 91.6%, SL EffNet is 95.5%, SL VGG16 is 95.2%, and proposed framework is 99.1%. This achieved an accuracy of the proposed framework that can be further verified by a confusion matrix, illustrated in Figure 7. The second highest accuracy is obtained by the Softmax classifier of 97.6% for the proposed framework. Furthermore, the computational time of each classifier for all middle steps is also noted, and it is observed that the ELM execution time is minimum than the rest of the classifiers. Moreover, it is also perceived that the selected feature execution time is almost half of the originally extracted deep features.

**Figure 8:**
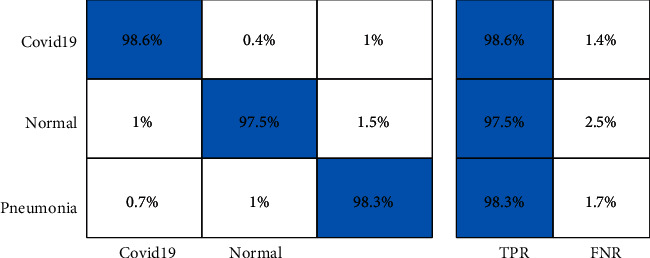
Confusion matrix of ELM classifier on Covid-GAN and Covid-Net mini Chest X-ray dataset using the proposed framework.

Table 3 presents the proposed framework classification results for Covid-GAN and Covid-Net mini Chest X-ray dataset. In this table, results are computed for all middle steps and compared with the entire proposed framework in terms of accuracy and time. ELM classifier gives better results of 93.3, 89.2, 95.8, 95.9, and 98.2% for EffNet, VGG16, SL EffNet, SL VGG16, and the proposed framework. The accuracy of the proposed framework that is 98.2% is further verified by a confusion matrix, given in Figure 8. In this figure, it is described that the COVID-19 class has the highest prediction rate of 98.6%. The classification accuracy of ELM is further compared with some other state-of-the-art classifiers such as Softmax, Naïve Bayes, and MCSVM. For these three classifiers, the best obtained accuracy is 97.2, 94.6, and 96.9%, respectively. The second highest accuracy is obtained by the Softmax classifier of 97.2% for the proposed framework. Furthermore, the computational time of each classifier for all middle steps and the entire proposed framework is also noted, and it is perceived that the execution time of ELM is less than the other listed classifiers.

**Figure 9:**
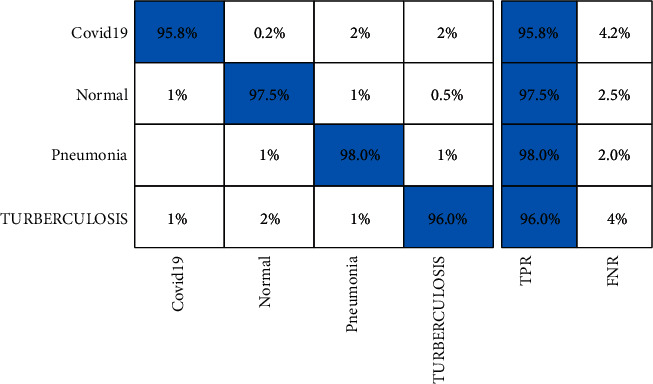
Confusion matrix of ELM classifier on Chest X-ray dataset using the proposed framework.

Table 4 describes the proposed framework classification results for the Chest X-ray dataset. In this table, results are computed for all middle steps and compared with the entire proposed framework in terms of accuracy and time. ELM classifier gives better results of 87.8, 84.1, 93.4, 92.8, and 96.7%, respectively, for EffNet, VGG16, SL EffNet, SL VGG16, and the proposed framework. The obtained accuracy of the proposed framework is 96.7% that is further verified by a confusion matrix, as presented in Figure 9. In this figure, it is described that the correct prediction rate of COVID-19 is 95.8%, whereas in other classes, such as normal, pneumonia, and tuberculosis, prediction rates are 97.5, 98.0, and 96.0%, respectively. The classification accuracy of ELM is further compared with some other state-of-the-art classifiers such as Softmax, Naïve Bayes, and MCSVM. For these three classifiers, the best obtained accuracy is 95.8, 93.4, and 94.8%, respectively. Based on these values, it is observed that the Softmax classifier has the second best accuracy after ELM. Furthermore, the computational time of each classifier for all middle steps and the entire proposed framework is also noted, and it is observed that ELM classifier execution time is minimum compared to the other listed classification methods.

### 4.2. Analysis

**Figure 10:**
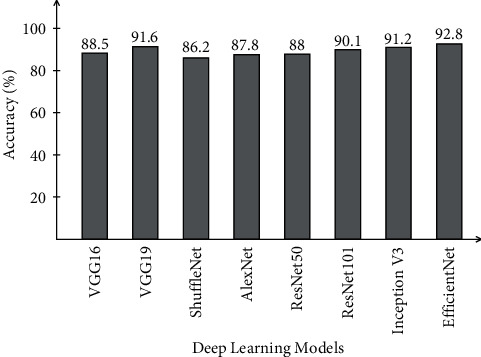
Comparison of different neural nets in terms of accuracy on COVID-19 Radiography database.

**Figure 11:**
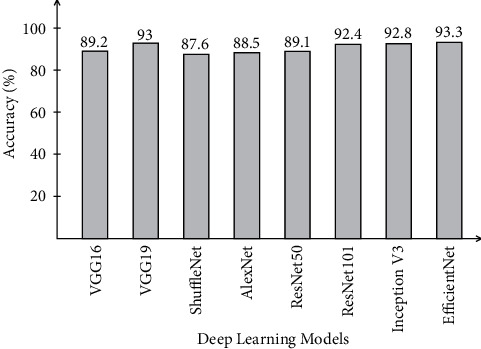
Comparison of different neural nets in terms of accuracy on Covid-GAN and Covid-Net mini Chest X-ray dataset.

**Figure 12:**
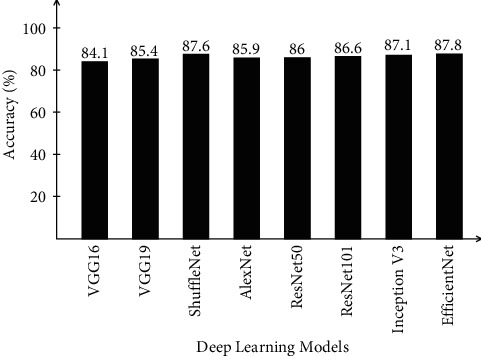
Comparison of different neural nets in terms of accuracy on Chest X-ray dataset.

A brief analysis of the proposed framework of COVID-19 classification using light weight deep learning models and explainable AI is presented here. The proposed framework was tested on three publicly available datasets and achieved better accuracy, as presented in Tables 2–4. The obtained accuracy of the ELM classifier for each dataset is further verified by a confusion matrix, as illustrated in Figures 7–9. The computational time of each classifier for all middle steps is also given in Tables 2–4. Based on the time and accuracy, it is shown that the ELM classifier performed better for the proposed framework. To further analyze the performance of lightweight deep models, a comprehensive comparison is conducted among several neural nets; accuracy is plotted in Figures 10–12. In Figure 10, the selected neural nets are tested on the COVID-19 Radiography database and achieve the highest accuracy of 92.8% on the EfficientNet model, whereas on the other models such as VGG16, VGG19, ShuffleNet, AlexNet, ResNet50, ResNet101, and InceptionV3, they obtained accuracies of 88.5, 91.6, 86.2, 87.8, 88, 90.1, and 91.2%, respectively. In Figure 11, the best achieved accuracy of 93.3% was for the EfficientNet deep model, whereas the rest of the models attained accuracies of 89.2, 93, 87.6, 88.5, 89.1, 92.4, and 92.8%, respectively. Similarly, In Figure 10, the selected neural nets are tested on Chest X-ray datasets, and EfficientNet attained the highest accuracy of 87.8%. Based on these accuracies, EfficientNet and VGG16 deep models are selected in this work for deep feature extraction.

## 5. Conclusions

COVID-19 has been a hot research topic in recent years due to a large number of deaths worldwide. Many computer-based techniques have been introduced by researchers, but there is still much room for improvement in terms of accuracy and computational time. We proposed a deep learning and explainable AI-based framework for COVID-19 diagnosis and classification using chest X-ray images in this paper. The proposed framework includes several steps, from contrast enhancement to ELM-based classification. The Grad-CAM-based visualization is used to highlight the image's hot spots. Three datasets were used in the experiment, and the results were significantly better than the middle steps and a few other neural nets. Based on the findings, it is concluded that the contrast enhancement and feature optimization techniques improve the proposed framework's accuracy. Furthermore, the optimization method reduced classification execution time. The static threshold value of the optimization algorithm is the work's limitation. More datasets will be considered for the experimental process in the future, and the selection process will be automated (threshold value).

## Figures and Tables

**Table 1:** Summary of selected datasets for the evaluation process.

Dataset	Classes	Images	Total images
COVID-19 Radiography database	COVID-19	3,616	1,345
	Lung opacity	6,012	
	Normal	10,192	
	Viral pneumonia	21,165	

Covid-GAN and Covid-Net mini Chest X-ray	Corona	461	4,481
	Normal	1,575	
	Pneumonia	6,517	

Chest X-ray (pneumonia, COVID-19, and tuberculosis)	COVID-19	566	6,897
	Normal	1,575	
	Pneumonia	4,265	
	Tuberculosis	491	

**Table 2:** Proposed framework COVID-19 classification results on COVID-19 Radiography database.

Classifiers	Features					Measures	
	EffNet	VGG16	SL EffNet	SL VGG16	Proposed	Accuracy (%)	Time (%)
Softmax	✓					90.1	122.8954
		✓				90.6	151.4584
			✓			95.2	78.5363
				✓		95.0	91.6678
					✓	97.6	70.7674

Naïve Bayes	✓					88.4	131.4453
		✓				88.9	162.5654
			✓			93.5	87.3422
				✓		93.8	97.0864
					✓	95.1	86.2355

MCSVM	✓					91.0	126.4433
		✓				91.5	149.5465
			✓			93.9	81.6743
				✓		94.5	97.7682
					✓	95.9	76.3476

ELM	✓					92.8	114.6752
		✓				88.5	136.8684
			✓			95.5	72.9005
				✓		95.2	86.0454
					✓	99.1	65.6294

**Table 3:** Proposed framework COVID-19 classification results on Covid-GAN and Covid-Net mini Chest X-ray dataset.

Classifiers	Features					Measures	
	EffNet	VGG16	SL EffNet	SL VGG16	Proposed	Accuracy (%)	Time (%)
Softmax	✓					91.6	91.4534
		✓				89.4	94.3423
			✓			94.8	62.2322
				✓		93.5	64.5454
					✓	97.2	47.6654

Naïve Bayes	✓					90.1	85.3843
		✓				87.5	78.6434
			✓			92.2	51.5444
				✓		90.9	49.9845
					✓	94.6	41.9905

MCSVM	✓					90.6	82.5645
		✓				90.1	78.9964
			✓			94.2	59.4354
				✓		92.9	64.8634
					✓	96.9	48.6654

ELM	✓					93.3	71.5535
		✓				89.2	69.6543
			✓			95.8	52.5454
				✓		95.9	47.6543
					✓	98.2	39.6652

**Table 4:** Proposed framework COVID-19 classification results on Chest X-ray dataset.

Classifiers	Features					Measures	
	EffNet	VGG19	SL EffNet	SL VGG19	Proposed	Accuracy (%)	Time (%)
Softmax	✓					86.3	94.8453
		✓				85.4	96.0985
			✓			91.6	69.3345
				✓		90.2	69.5884
					✓	95.8	53.9484

Naïve Bayes	✓					84.2	89.2322
		✓				85.7	85.9505
			✓			88.3	57.2754
				✓		89.6	62.9576
					✓	93.4	59.2328

MCSVM	✓					85.6	88.5445
		✓				85.0	86.6544
			✓			89.9	62.8432
				✓		90.0	67.4454
					✓	94.8	53.4935

ELM	✓					87.8	74.5563
		✓				84.1	71.0054
			✓			93.4	54.5374
				✓		92.8	51.0588
					✓	96.7	43.2084

## Data Availability

The proposed framework is evaluated on three publically Kaggle COVID datasets such as COVID-19 Radiography dataset (https://www.kaggle.com/tawsifurrahman/covid19-radiography-database), Covid-GAN and Covid-Net mini Chest X-ray dataset (https://www.kaggle.com/yash612/covidnet-mini-and-gan-enerated-chest-xray), and Chest X-ray dataset (https://www.kaggle.com/jtiptj/chest-xray-pneumoniacovid19tuberculosis), respectively.
